# CXCL13 chemokine is a novel player in multiple myeloma osteolytic microenvironment, M2 macrophage polarization, and tumor progression

**DOI:** 10.1186/s13045-022-01366-5

**Published:** 2022-10-10

**Authors:** Katia Beider, Valeria Voevoda-Dimenshtein, Ali Zoabi, Evgenia Rosenberg, Hila Magen, Olga Ostrovsky, Avichai Shimoni, Lola Weiss, Michal Abraham, Amnon Peled, Arnon Nagler

**Affiliations:** 1 Division of Hematology and CBB, Chaim Sheba Medical Center, Tel Aviv University, Tel-Hashomer, Israel; 2 Goldyne Savad Institute of Gene Therapy, Hebrew University Hospital, Jerusalem, Israel

## Abstract

**Background:**

We assessed the mechanism by which multiple myeloma (MM) shapes the bone marrow (BM) microenvironment and affects MΦ polarization.

**Methods:**

In vivo xenograft model of BM-disseminated human myeloma, as well as analysis of MM cell lines, stromal components, and primary samples from patients with MM, was utilized.

**Results:**

Analysis of the BM from MM-bearing mice inoculated with human CXCR4-expressing RPMI8226 cells revealed a significant increase in M2 MΦ cell numbers (*p* < 0.01). CXCL13 was one of the most profoundly increased factors upon MM growth with increased levels in the blood of MM-bearing animals. Myeloid cells were the main source of the increased murine CXCL13 detected in MM-infiltrated BM. MM cell lines induced CXCL13 and concurrent expression of M2 markers (MERTK, CD206, CD163) in co-cultured human MΦ in vitro. Interaction with MΦ reciprocally induced CXCL13 expression in MM cell lines. Mechanistically, TGFβ signaling was involved in CXCL13 induction in MM cells, while BTK signaling was implicated in MM-stimulated increase of CXCL13 in MΦ. Recombinant CXCL13 increased RANKL expression and induced TRAP+ osteoclast (OC) formation in vitro, while CXCL13 neutralization blocked these activities. Moreover, mice inoculated with CXCL13-silenced MM cells developed significantly lower BM disease. Reduced tumor load correlated with decreased numbers of M2 MΦ in BM, decreased bone disease, and lower expression of OC-associated genes. Finally, higher levels of CXCL13 were detected in the blood and BM samples of MM patients in comparison with healthy individuals.

**Conclusions:**

Altogether, our findings suggest that bidirectional interactions of MΦ with MM tumor cells result in M2 MΦ polarization, CXCL13 induction, and subsequent OC activation, enhancing their ability to support bone resorption and MM progression. CXCL13 may thus serve as a potential novel target in MM.

**Supplementary Information:**

The online version contains supplementary material available at 10.1186/s13045-022-01366-5.

## Introduction

Multiple myeloma (MM) is a B-cell neoplasm characterized by clonal expansion of malignant plasma cells in the bone marrow (BM) compartment, where they proliferate and acquire resistance to chemotherapy-mediated apoptosis. MM accounts for 10% of malignant hematological diseases [1, 2]. The outcome for patients with MM has considerably improved over the last two decades with the incorporation of novel agents including proteasome inhibitors, immunomodulatory drugs (IMiDs), and anti-CD38 antibodies [3–5]. Nevertheless, despite the advances in current therapy MM remains incurable due to the development of drug resistance, which manifests as relapsed/refractory disease [6]. As signals from the tumor microenvironment are known to make pivotal contributions to the progression of hematopoietic and epithelial malignancies, increasing emphasis is now being placed on targeting the tumor cell microenvironment. The main challenge is dissecting the complex cellular and molecular interactions between tumor cells and the host. Interaction of malignant plasma cells with the BM microenvironment is critical for homing, survival, and drug-resistance acquisition of MM in the BM niche [2, 7]. The BM milieu contains various components, including stromal cells (BMSCs) as well as osteoclasts and immune cells. BMSCs are known to promote growth and drug resistance in MM cells [8]. However, the functional role of other components of the microenvironment is less clear. Reciprocal positive and negative interactions between plasma cells and BM stroma are orchestrated by an array of cytokines, receptors, and adhesion molecules [9], suggesting that both myeloma-derived and stromal cell-produced factors, such as chemokines, participate in the regulation of MM development and progression [10]. Different chemokines not only regulate the homing and re-circulation of MM cells but also enhances tumor growth, vascularization, and bone destruction [11, 12]. Chemokine (C-X-C motif) ligand 13 (CXCL13) is normally expressed in secondary lymphoid organs by follicular dendritic cells, macrophages, and fibroblasts [13–15]. CXCL13 exerts its functions through its cognate receptor, C-X-C chemokine receptor type 5 (CXCR5), which is expressed on mature B cells and subsets of follicular helper T cells and regulatory T cells [16]. CXCL13 is essential for B cell homing into lymphoid tissue and is required for embryonic development of the majority lymph nodes and is involved in autoimmunity and inflammatory conditions [14, 17–19]. Furthermore, recent studies implicate the cancer-promoting role of dysregulated CXCL13-CXCR5 chemokine pathway in various hematologic [20–22] and solid malignancies [23–25], while no data are available in MM.

Here, we identify CXCL13 as being a novel factor involved in MM pathogenesis. We demonstrate that CXCL13 is strongly upregulated in the MM BM microenvironment and is associated with MM-related niche changes, supporting M2 macrophage polarization and promoting osteoclast activation. Studying the underlying mechanisms of CXCL13 induction and its role in MM disease progression may help to develop new therapeutic targets and new biomarkers in MM.

## Materials and methods

### MM cell lines

The following human MM cell lines were obtained from ATCC (Rockville, MD, USA): RPMI8226, U266, and NCI-H929. The CAG MM cell line (generated by the group at the University of Arkansas for Medical Sciences (UAMS) [26]), OPM-1, and OPM-2 (originate from the same individual) were kindly provided by Prof. Israel Vlodavsky, Technion, Israel. Cells were maintained in log-phase growth in RPMI1640 medium (Biological Industries) supplemented with 10% heat-inactivated fetal calf serum (FCS), 1 mM L-glutamine, 100 U/ml penicillin, 0.01 mg/ml streptomycin, and 1 mM sodium pyruvate (Biological Industries) in a humidified atmosphere of 5% CO_2_ at 37 °C. U266 cells were authenticated in 2017 by short tandem repeat (STR) DNA profiling using AmpFISTR Identifier Kit (Applied Biosystems) and other cell lines were authenticated in 2021 at the Genomics Center of Biomedical Core Facility, Technion using the Promega GenePrint 24 System.

### MM patient samples

Primary MM cells were isolated from bone marrow aspirates of consenting myeloma patients. Six normal BM samples served as controls. The study was approved by the institutional review board of the Sheba Medical Center. Mononuclear cells were collected after standard separation on Ficoll-Paque (Pharmacia Biotech) and cryopreserved in liquid nitrogen. Bone marrow plasma was separated by centrifugation and stored in aliquots at -80 °C. Peripheral blood plasma samples were collected from 61 MM patients and 9 healthy volunteers and stored in aliquots at − 80 °C.

### Inhibitors

The following chemicals were used: bortezomib, ibrutinib, acalabrutinib, zanubrutinib and SB-431542 purchased from Cayman, ST2825 purchased from APExBIO. Lipopolysaccharide (LPS) was purchased from Sigma-Aldrich.

### CXCR4 overexpression

To stably over-express CXCR4, RPMI8226 cells were transduced with the lentiviral bicistronic vector encoding for CXCR4 and GFP genes, as previously described [27].

### CXCL13 silencing using CRISPR/Cas9 lentiviral system

CXCL13 gene in RPMI8226-CXCR4-GFP cells was silenced using pLenti-U6-sgRNA-SFFV-Cas9-2A-Puro lentiviral vectors (ABM), constructed against three different target sequences (TCTATTACACAAGCTTG, ATTCAAATCTTGCCCCG, CAAGTCAATTGTGTGTG). Briefly, the cells were stably transduced with lentiviral vectors using a three-plasmid system: anti-CXCL13 plasmids, envelope coding plasmid VSV-G, and a packaging construct CMVDR8.91.

### Murine xenograft models of disseminated human MM

NSG mice were maintained under defined flora conditions at the Hebrew University Pathogen-Free Animal Facility (Jerusalem, Israel). All experiments were approved by the Animal Care Committee of the Hebrew University. Mice were injected intravenously with RPMI8226-EV, RPMI8226-CXCR4-GFP, or RPMI8226-CXCR4-GFP-CRISPR-CXCL13 human cells (5 × 10^6^/mouse) and were killed 24 days after tumor inoculation. The disease was verified by measurement of human immunoglobulin in the plasma of inoculated mice using the ELISA kit (Immunology Consultants Laboratory). Bone marrow-derived cells were obtained from the femurs and tibias of inoculated animals by flushing with phosphate-buffered saline. To investigate the therapeutic potential of bortezomib, three days after inoculation with RPMI8226-CXCR4-GFP cells, mice were randomized and treated with intraperitoneal (i.p.) injections of bortezomib (1 mg/kg) twice per week, for a total of 6 injections. Animals were killed 24 days after tumor inoculation.

### Cell migration assay

Migration assay was performed in triplicates using 5-µm pore size transwells (Costar). The lower compartment was filled with 600 µl of 1% FCS RPMI 1640 medium containing CXCL12 (200 ng/ml) (PeproTech EC), and 5 × 10^5^ MM cells in 100 µl of 1% FCS RPMI1640 medium were applied to the upper compartment. The number of cells migrating within 4 h to the lower compartment was determined by FACS and expressed as a percentage of the input.

### Cytokine array

Murine cytokine plasma levels were determined semi-quantitatively using the Proteome Profiler Mouse Cytokine Array (ARY006, R&D systems, Minneapolis, MN), which contains 4 membranes and each spotted in duplicate with 40 different cytokine antibodies. Peripheral blood plasma from non-inoculated (*n* = 3) and RPMI8226-CXCR4-GFP-inoculated (*n* = 3) animals were collected and pooled and the array was performed according to the manufacturer's instructions. The blots were imaged using a Chemidoc system from Bio-Rad. Densitometric analysis was performed with ImageLab software.

### CXCL13 immunohistochemistry

Commercial MM BM tissue microarray containing ten cases of myeloma, ten cases of plasmacytoma and two normal BM tissue samples was used (US Biomax). For histologic analysis, the bone specimens from myeloma-inoculated mice were fixed in freshly prepared 4% paraformaldehyde, decalcified in 10% EDTA with 0.5% paraformaldehyde, and embedded in paraffin using standard procedures. Paraffin-embedded sections were initially dewaxed, rehydrated, and subjected to heat-induced epitope retrieval using Antigen Retrieval Reagent-Basic (R&D Systems). Samples were then incubated overnight at 4 °C in a humidified chamber with anti-human or anti-mouse CXCL13 antibodies (R&D Systems) diluted to a final concentration of 15 µg/mL. Next, the sections were incubated with secondary anti-goat horseradish peroxidase-conjugated antibody (DakoCytomation) for 30 min at room temperature. 3-Amino-9-ethyl carbazole (AEC) was used for color development, and the sections were counterstained with hematoxylin.

### CXCL13, TGFβ, and RANKL ELISA

CXCL13 levels in conditioned medium, BM, and peripheral blood plasma from MM patients and healthy volunteers were examined using a human CXCL13 ELISA kit (RayBiotech) according to the manufacturer's instructions. TGFβ levels in conditioned medium were examined using a human TGFβ ELISA kit (R&D Systems) according to the manufacturer's instructions. The murine RANKL levels in the plasma of non-inoculated (*n* = 3), RPMI8226-CXCR4-GFP-inoculated (*n* = 4) or RPMI8226-CXCR4-GFP-CRISPR-CXCL13-inoculated (*n* = 4) animals were determined using murine RANKL ELISA kit (R&D systems) according to the manufacturer's instructions.

### Generation of osteoclasts

Osteoclast multinucleated cells were generated in vitro. Peripheral blood mononuclear cells (PBMCs) obtained from healthy donors were collected after standard separation on Ficoll-Paque (Pharmacia Biotech). Adherent cells were prepared from PBMCs and cultured in 24-well plates in Eagle minimal essential medium alpha modification (alpha-MEM; GIBCO) supplemented with 10% heat-inactivated FCS (Biological Industries), 100 ng/mL soluble RANK ligand (RANKL), and 100 ng/mL M-CSF (Peprotech) for 7 days. Media were replenished twice a week. Alternatively, adherent PBMCs were treated with 500 ng/mL CXCL13 (R&D Systems) for 14 days, in the absence or presence of 10 µg/mL neutralizing anti-CXCL13 antibodies (R&D Systems).

### TRAP staining

Tartrate-resistant acid phosphatase (TRAP) staining was performed to detect osteoclast differentiation ability. The cells were fixed in 4% paraformaldehyde for 20 min and stained using the TRAP kit (Sigma-Aldrich) according to the manufacturer’s instructions. Osteoclasts were determined to be TRAP-positive staining multinuclear (> 3 nuclei) cells by light microscopy. The total number of TRAP-positive cells in each well was counted.

### Preparation of bone marrow stromal cells, macrophages, and co-culture experiments

Primary human bone marrow stromal cells (BMSCs) were generated from bone marrow aspirates of consenting healthy donor volunteers. BMSCs were isolated by plate adherence and expanded as previously described [28]. For macrophage generation, PBMCs were obtained from the blood of consenting healthy donor volunteers by Ficoll-Paque density centrifugation and allowed to adhere to culture plates for 2 h at 37 °C. Non-adherent lymphocyte-enriched cells were removed. The adherent monocytes were incubated for 10 days in DMEM medium supplemented with 10% heat-inactivated FCS, 100 U/ml penicillin, 100 µg/ml streptomycin, 2 mM *L*-glutamine, and 1 mM sodium pyruvate. The medium was changed and non-adherent cells were discarded every two days. The purity of monocyte-derived cells was verified by flow cytometry using the CD14 marker expression analysis and was > 95%. For co-culture, MM cells were either seeded on top of the stromal cells or separated with 0.4 µm pore transwells (Costar). Following 48 h, cells and conditioned medium were collected for subsequent analysis.

### Western immunoblot analysis

Total protein lysates (50 μg) were resolved by electrophoresis in 10% SDS-PAGE and transferred onto PVDF membranes. Blots were subjected to a standard immuno-detection procedure using specific antibodies and the ECL substrate (Biological Industries). The signal was detected using a Bio-Rad image analyzer (Bio-Rad) and analyzed using ImageLab software. The primary antibodies used were: phospho-cJun, phospho-JNK, phospho-p38, phospho-STAT3 (Cell Signaling Technology), and β-actin (Sigma-Aldrich).

### RT-PCR analysis

Total RNA from murine BM cells, BM samples from patients with MM, or cultured MM cell lines was extracted using Trizol reagent (Invitrogen) according to the manufacturer’s instructions. To generate cDNA, 1 μg total RNA was reverse-transcribed using the qScript cDNA Synthesis Kit (Quanta) according to the manufacturer's instructions. Real-time quantitative PCR (RT-qPCR) was performed in a final volume of 20 μL, containing 100 ng of total RNA-derived cDNAs, forward and reverse primers (300 nM), and PerfeCta SYBR Green FastMix (Quanta Biosciences), using the StepOnePlus Real-Time PCR system (Applied Biosystems). Changes in expression levels were normalized to control β2-microglobulin using the ΔΔ*C*_T_ method of relative quantification using the StepOne Software v2.2. Experiments were performed in triplicates for each sample. For murine genes, changes in expression levels were normalized to control HPRT. The sequences of primers are presented in Additional file 1: Table S1.

### Flow cytometry analysis

The expression of CD11b, F4/80, CD206, and MERTK on the surface of murine BM mononuclear cells was evaluated using specific monoclonal antibodies. Expression of CD206, CD163, and MERTK on human macrophages cultured alone or in the presence of MM cells and in primary MM BM samples was evaluated by co-staining with CD11b. The frequency of myeloid cell populations in the primary MM BM samples was evaluated by co-staining for CD11b, CD14 and CD16 markers. The frequency of osteoclast precursors in the primary MM BM samples was evaluated by co-staining for CD11b, RANK, and CD51 markers. All antibodies including appropriate isotype control were purchased from Biolegend and are listed in Additional file 1: Table S2. Data were acquired using Navios Beckman Coulter instrument and analyzed with Kaluza Analysis Software version 2.

### Statistical analyses

Data are expressed as the mean ± standard deviation (STDEV) or standard error (SE). Statistical comparisons of means between two groups were made by a two-tailed unpaired Student's *t-test*.

## Results

### M2-like macrophages (MΦ) are increased in MM-infiltrated bone marrows from the murine xenograft model of disseminated MM

**Fig. 1:**
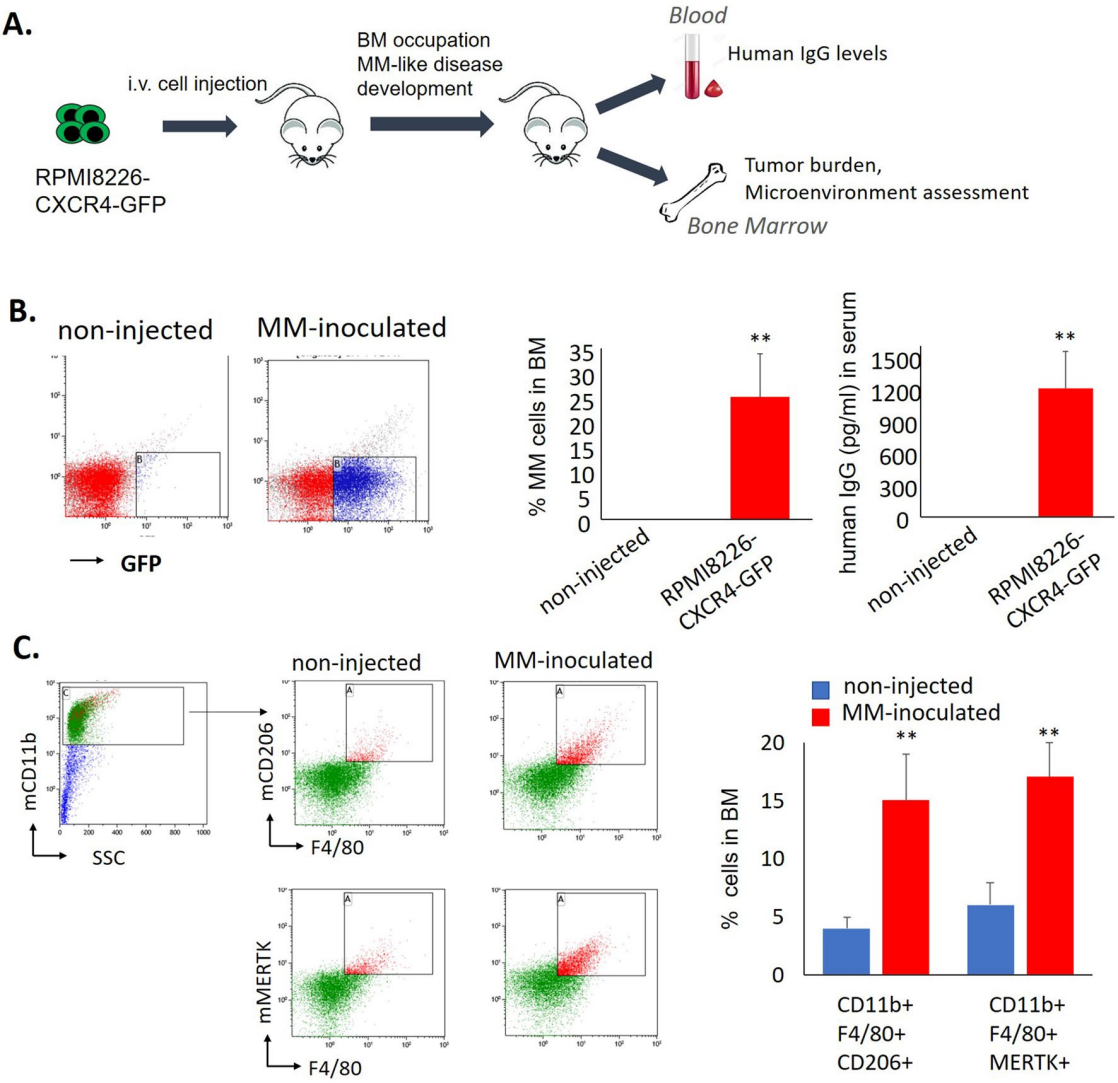
M2 macrophages are increased in BM of MM-inoculated mice. **A** Schematic representation of xenograft systemic model of CXCR4-driven MM with BM involvement. NSG mice were i.v. inoculated with RPMI8226-CXCR4-GFP cells. Non-injected NSG mice were used as controls. **B** MM tumor load was measured by enumeration of GFP+ human MM cells in the BM and by evaluation of human IgG levels in the blood of non-injected (*n* = 5) or inoculated animals (*n* = 5). Data are presented as mean ± SE, **p < 0.01. **C** Presence of M2 murine MΦ in the BM of non-injected controls (*n* = 5) or MM-inoculated animals (*n* = 5), evaluated by flow cytometry. Representative plots for gating strategy evaluating the expression of murine CD11b, F4/80, CD206, and MERTK markers. Bars showing mean of triplicates ± STDEV, **p < 0.01

Previously, we described our newly established xenograft mouse model of disseminated MM with BM involvement, based on i.v. injection of CXCR4-overexpressing RPMI8226 cells into immune-compromised NSG mice [27]. Lethal disease with MM characteristics and preferential BM localization of human GFP-expressing MM cells was developed (Fig. 1A, B). To assess the changes in the BM milieu associated with MM development, we analyzed the myeloid composition of murine BM occupied with human MM cells. Mice were killed on day 24 after the inoculation with human MM cells (end-stage of disease) and compared with healthy non-injected NSG mice. A general CD11b-positive monocytic/macrophage population was gated for the analysis, macrophage discrimination was based on the expression of the F4/80 marker. For M2-like macrophage detection, we used CD206 and MERTK-specific markers. A significant increase in CD206+ MERTK+ M2-like macrophages was detected in BM of MM-bearing animals in comparison with non-injected controls (*p* < 0.01) (Fig. 1C).

### Murine CXCL13 is elevated in sera of MM-inoculated mice and BM macrophages

**Fig. 2:**
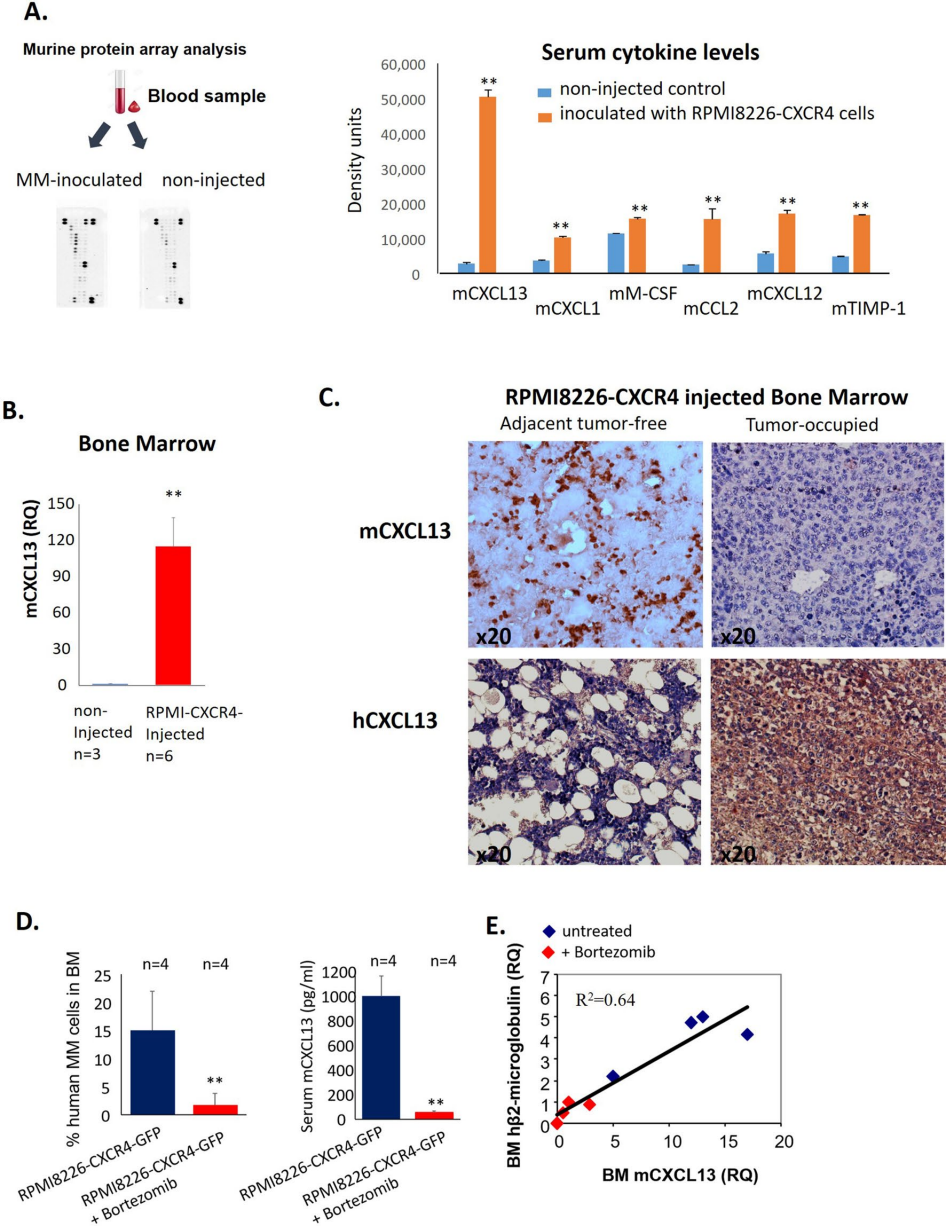
Murine CXCL13 is increased in the blood and BM of MM-inoculated mice. **A** Evaluation of cytokine levels in the serum of MM-inoculated (pooled from 3 mice) and non-injected control (pooled from 3 mice) animals using cytokine array. **B** Levels of mCXCL13 in the BM of MM-inoculated (*n* = 6) and control mice (*n* = 3), evaluated by qRT-PCR. Data are presented as the mean of triplicates ± STDEV (**p < 0.01). **C** Immunohistochemistry for mCXCL13 and human CXCL13. **D**, **E** RPMI8226-CXCR4-GFP cells were injected i.v. into NSG mice. Mice were either untreated (control group, *n* = 4) or treated with subcutaneous injections of bortezomib (1 mg/kg, twice a week) (*n* = 4). **D** Tumor burden (%GFP + cells) in BM and serum levels of murine CXCL13 were evaluated on day 24. **E** Co-expression of human β2-microglobulin and murine *Cxcl13 *in the BM of MM-inoculated mice, evaluated by qRT-PCR

MM-related enrichment in M2-like macrophages in the BM niche can result in alterations in cytokine expression levels, either locally, in the BM milieu, or systemically. We thus evaluated the levels of 40 murine cytokines and chemokines in the serum of MM-bearing mice compared to healthy non-injected controls using a commercial cytokine array. Several factors were found to be significantly elevated in peripheral blood of animals with progressive MM disease, including mouse CCL2, CCL5, CXCL1, and CXCL12 chemokines as well as M-CSF cytokine and TIMP-1. Notably, the most profoundly increased factor among 40 cytokines analyzed with array was CXCL13 chemokine (Fig. 2A).

Next, to address the question of whether increased blood levels of mouse CXCL13 reflected the changes in the BM myeloid compartment induced by MM disease, we examined the levels of murine CXCL13 transcript in BM of MM-bearing animals. Significantly upregulated levels of *Cxcl13* mRNA (measured by qRT-PCR) were detected in MM-infiltrated BMs in comparison with non-injected controls (Fig. 2B). Our next goal was to identify the cell source of increased CXCL13. Therefore, we performed an immunohistochemical analysis of tissue sections from MM-occupied murine femur bones affected by MM. As we previously reported [27], intravenous injection of RPMI8226-CXCR4 cells results in their preferential BM localization and development of disease with features consistent with those of MM in human patients, including the patchy pattern of distribution, with foci of human myeloma cells surrounded by adjacent murine BM tissue. Therefore, we analyzed the expression of murine CXCL13 in BM tissue of tumor-bearing mice. As can be seen in Fig. 2C, host myeloid cells are the main source of the increased murine CXCL13 seen in the areas adjacent to MM-produced tumors (Fig. 2C upper panel). Importantly, RPMI8226-CXCR4 myeloma cells were found to express high levels of human CXCL13 (Fig. 2C lower panel).

Subsequently, we evaluated murine CXCL13 levels following anti-myeloma therapy with bortezomib. Reduction of the MM load upon treatment with bortezomib resulted in a corresponding decrease in murine CXCL13 serum levels (Fig. 2D). Moreover, murine *Cxcl13* mRNA expression strongly correlated with human β2-microglobulin mRNA levels in BM (*p* < 0.0001 *R*^2^ = 0.64), indicating the interrelation between tumor burden and CXCL13 induction (Fig. 2E). These results suggest the possible utilization of CXCL13 levels as a surrogate marker for response to anti-MM treatments.

### CXCL13 is up-regulated upon the cross talk between MM cells and cellular components of the MM microenvironment

**Fig. 3:**
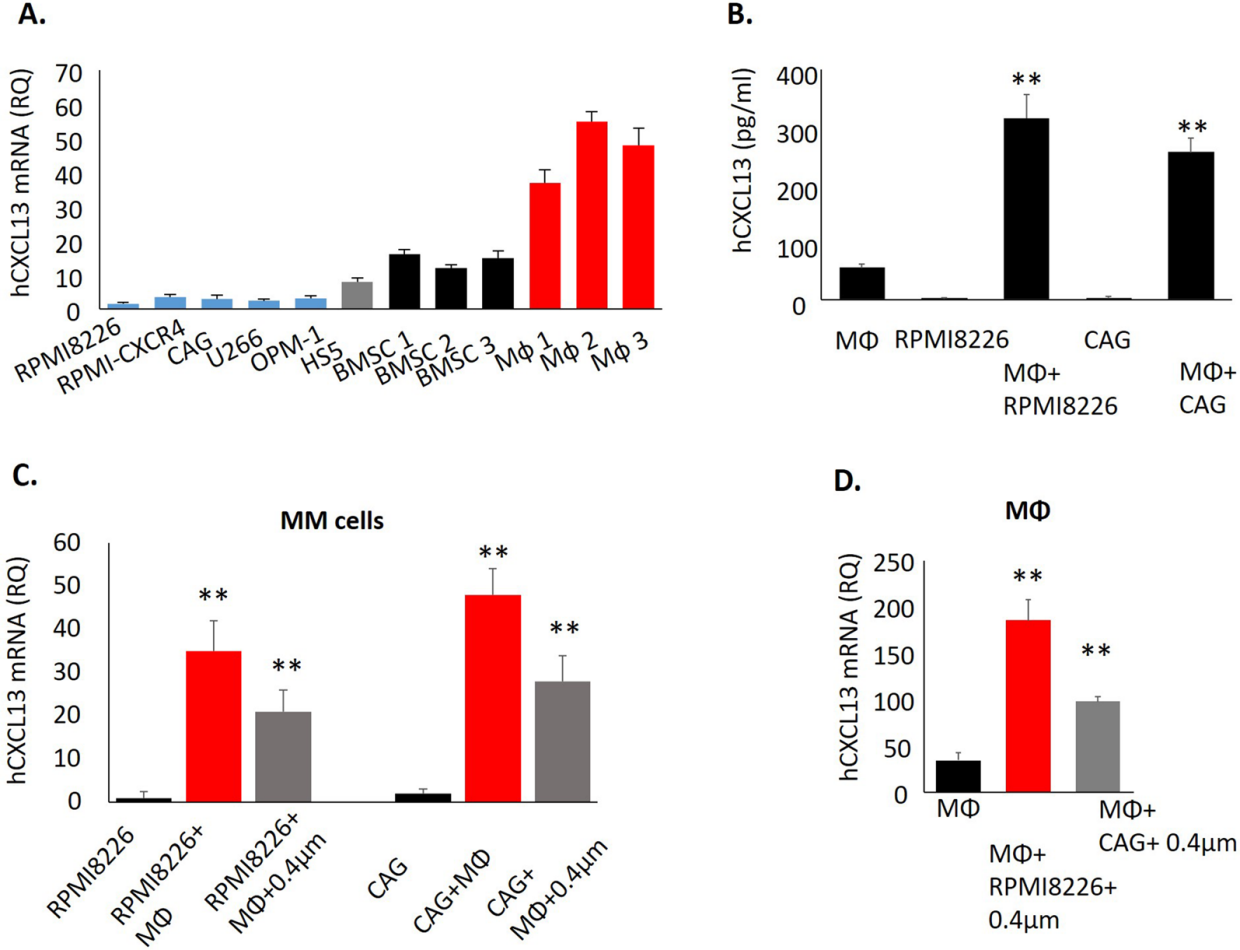
Interaction between MM cells and macrophages up-regulates CXCL13 in both cell populations. **A** CXCL13 mRNA expression in human MM cell lines, primary BMSC, and peripheral-blood generated MΦ, evaluated by qRT-PCR. Data are presented as the mean of triplicates ± STDEV (**p < 0.01). **B** Peripheral-blood-derived MΦ were cultured in the absence or presence of MM cell lines RPMI8226 or CAG (direct co-culture) for 48 h. Levels of secreted CXCL13 in the culture medium were evaluated by ELISA. Data are presented as the mean of triplicates ± STDEV (**p < 0.01). **C** CXCL13 mRNA levels in MM cells RPMI8226 and CAG, cultured in the absence or presence of peripheral-blood derived MΦ, either in direct contact co-culture or separated by 0.4 µm membrane, evaluated by qRT-PCR. **D** CXCL13 mRNA levels in peripheral-blood generated MΦ, cultured in the absence or presence of RPMI8226 and CAG cells, separated by a 0.4 µm membrane, evaluated by qRT-PCR. Data are presented as the mean of triplicates ± STDEV (**p < 0.01)

Next, CXCL13 transcript levels were analyzed in human MM cell lines (*n* = 5), human BM stromal cell line HS5, primary human BM stromal cells (BMSCs) (*n* = 3), and peripheral blood-generated macrophages (MΦ) (*n* = 3). As depicted in Fig. 3A, MM cell lines expressed a relatively low basal level of CXCL13, while significantly higher levels of CXCL13 were detected in BMSCs (mRNA RQ 8–16) and macrophages (mRNA RQ 37–55). We next measured secreted CXCL13 levels in a conditioned medium obtained from MM cells (RPMI8226 and CAG) and macrophages, grown either separately or co-cultured for 48 h. CXCL13 secretion was significantly increased upon the co-culture of MM cells with macrophages (Fig. 3B). To identify the source of elevated CXCL13 following co-incubation, we assessed CXCL13 gene expression in the MM cells, cultured either alone or co-cultured with macrophages. Notably, interaction with macrophages significantly induced CXCL13 expression in MM cells, in both direct co-culture as well as in trans-well separated cultures (Fig. 3C). These findings disclose the role of both contact-dependent and soluble factors for CXCL13 regulation in MM cells. In addition, significant up-regulation in CXCL13 gene expression was also detected in the macrophages cultured with an MM-produced conditioned medium, indicating MM cells provide stimulatory signals that reciprocally induce CXCL13 expression in macrophages (Fig. 3D). These results suggest that MM-macrophage cross talk reciprocally induces CXCL13 expression in both MM tumor and myeloid stromal cells.

### MM-induced up-regulation of CXCL13 in macrophages involves BTK signaling

**Fig. 4:**
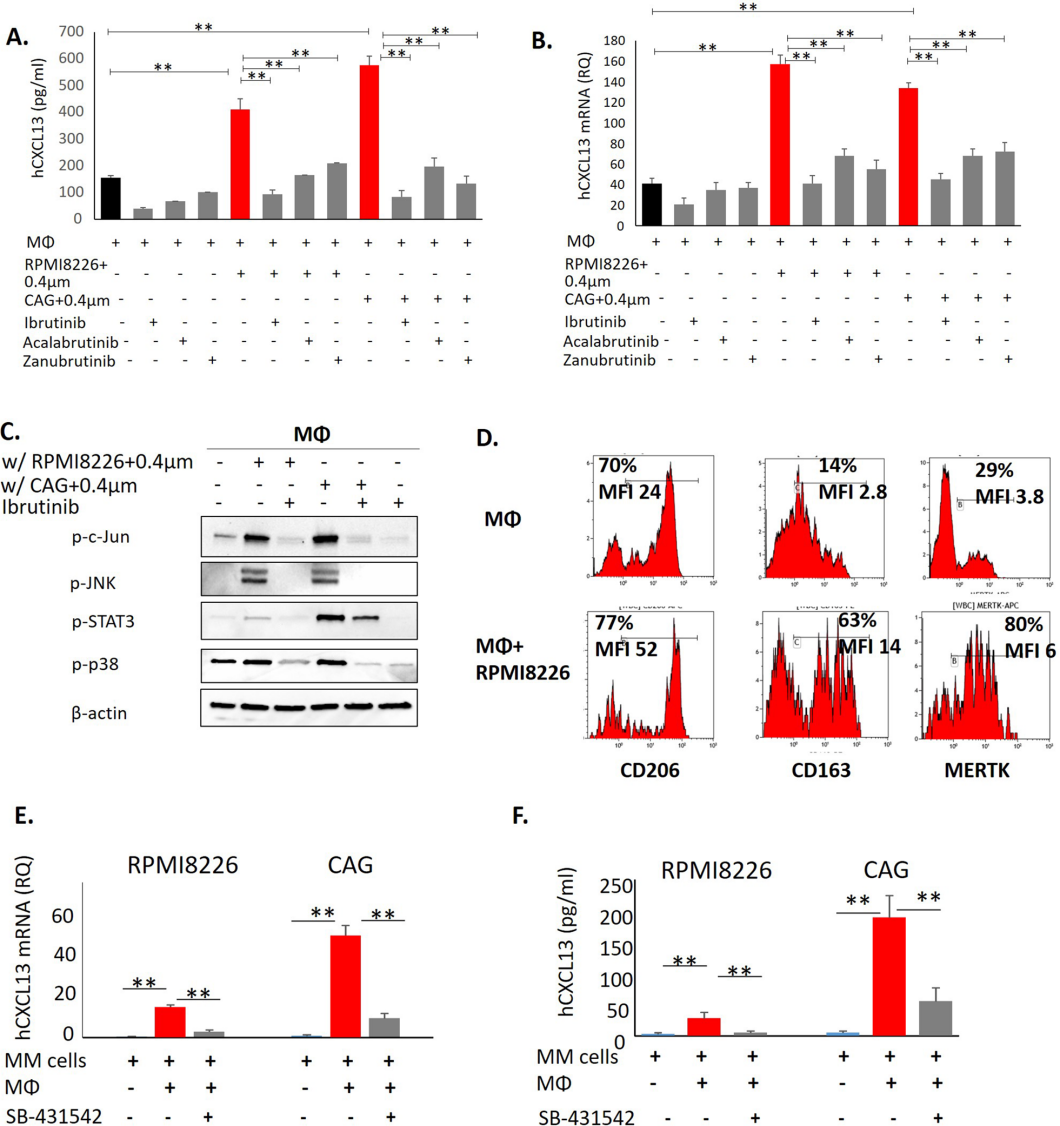
An increase in CXCL13 upon the interaction between MM cells and macrophages is mediated by BTK and TGFβ signaling. **A** Peripheral-blood-derived MΦ were cultured in the absence or presence of MM cells RPMI8226 (direct co-culture) with or without ibrutinib (20 µM), acalabrutinib (20 µM) and zanubrutinib (20 µM) for 48 h. The levels of secreted CXCL13 in the culture medium were evaluated by ELISA. Data are presented as the mean of triplicates ± STDEV (**p < 0.01). **B** Peripheral-blood derived MΦ were cultured in the absence or presence of MM cell lines RPMI8226 and CAG, separated by 0.4 µm membrane with or without ibrutinib (20 µM), acalabrutinib (20 µM) and zanubrutinib (20 µM) for 48 h and subjected to subsequent analysis. Expression levels of CXCL13 mRNA in MΦ cells evaluated by qRT-PCR. **C** MM-induced signaling in MΦ co-cultured in 0.4 µm transwells for 48 h, evaluated by Western blot analysis. Blots were probed for p-c-Jun, p-JNK, p-STAT3 and p-p38. β-actin was used as an internal control. Representative data from at least two independent experiments are shown. **D** Peripheral-blood-derived MΦ were cultured in the absence or presence of MM cells RPMI8226 (direct co-culture) for 48 h and subjected to flow cytometry analysis. Expression levels of cell surface CD206, CD163, and MERTK on gated CD11b+ cells are depicted in representative histograms. Quantification of geometric mean fluorescence intensity (MFI) of surface markers and percentage of positive cells was performed. **E** CXCL13 mRNA levels in MM cells RPMI8226 and CAG, cultured in the absence or presence of peripheral-blood derived MΦ, treated with SB-431542 (20 µM) for 48 h, evaluated by qRT-PCR. **F** Peripheral-blood-derived MΦ were cultured in the absence or presence of MM cells RPMI8226 and CAG (direct co-culture) with or without SB-431542 (20 µM) for 48 h. Levels of secreted CXCL13 in the culture medium were evaluated by ELISA. Data are presented as the mean of triplicates ± STDEV (**p < 0.01)

We next explored the signaling pathways involved in MM-mediated induction of CXCL13 in macrophages. Previous studies demonstrated that inhibition of Bruton’s tyrosine kinase (BTK) using ibrutinib suppresses macrophage activation and CXCL13 production [29]. In agreement, ibrutinib treatment significantly inhibited basal CXCL13 secretion in macrophages. Moreover, ibrutinib nearly completely abrogated the increase in CXCL13 induced by the co-culture of macrophages with RPMI8226 and CAG MM cells (Fig. 4A). Selective second-generation BTK inhibitors acalabrutinib and zanubrutinib demonstrated similar activity (Fig. 4A). Accordingly, BTK inhibition by ibrutinib, acalabrutinib and zanubrutinib effectively suppressed MM-mediated induction of CXCL13 mRNA levels in macrophages co-cultured with MM cells (Fig. 4B). These findings suggest that BTK signaling is involved in CXCL13 gene induction in macrophages upon activation by MM cells. Of note, BTK inhibition using ibrutinib in MM cells promoted only a weak reduction in CXCL13 gene expression, suggesting alternative signaling pathways mediating stroma-induced CXCL13 up-regulation in MM cells (Additional file 1: Fig. S1). Since BTK plays an important role in multiple signaling pathways, including toll-like receptor (TLR) signaling, the possible involvement of TLR4 in MM-induced CXCL13 induction in macrophages was assessed. TLR4 activation with LPS resulted in CXCL13 induction that was blocked by ibrutinib and ST2825, a specific inhibitor of TLR4 adaptor protein MYD88 (Additional file 1: Fig. S2). Importantly, interaction with MM cells accordingly induced TLR4 stimulation, since MYD88 inhibition effectively abrogated MM-mediated CXCL13 up-regulation in MΦ (Additional file 1: Fig. S2). These results suggest the upstream role of TLR4 and MYD88 activation in myeloma-induced BTK-dependent CXCL13 up-regulation in the macrophages.

To further explore the impact of MM-mediated induction of BTK in macrophages, downstream intracellular pathways were evaluated. Profound activation of BTK-regulated pathways was observed in the macrophages co-cultured with MM cells in trans-well experiments, including an increase in c-Jun, JNK, STAT3, and p38 phosphorylation. However, BTK inhibition using ibrutinib completely abrogated MM-induced signaling in the macrophages (Fig. 4C). MM cells are known to be involved in M2-like macrophage polarization [30, 31]. Indeed, we observed strong up-regulation of M2-associated markers in macrophages co-cultured with MM cells (Fig. 4D). Therefore, increased CXCL13 production in macrophages may reflect M2 phenotype acquisition upon the contact with MM cells. Altogether, these results indicate that MM cells up-regulate CXCL13 macrophage production in a BTK-dependent manner, while BTK inhibition with ibrutinib alleviates these pro-inflammatory responses in macrophages.

### Macrophage-induced up-regulation of CXCL13 in MM cells is mediated by TGFβ signaling

Next, we explored the signals that up-regulate CXCL13 in MM cells upon their interaction with stromal components. M2-polarized macrophages have been demonstrated to support tumor progression and affect the tumor microenvironment by producing multiple cytokines and growth factors, including TGFβ. In line with these findings, TGFβ secretion was significantly increased in the conditioned medium of macrophages co-cultured with MM cells (Additional file 1: Fig. S3A). Furthermore, up-regulation of TGFβ gene expression in M2-polarized macrophages upon interaction with MM cells was confirmed by qRT-PCR (Additional file 1: Fig. S3B). Based on these observations, and taking into account the known pro-myeloma role of macrophage-produced TGFβ, we evaluated the possible involvement of TGFβ signaling in macrophage-mediated induction of CXCL13 in MM cells. Inhibition of TGFβ activity using specific TGFβR1 inhibitor SB-431542 significantly reduced the secretion of CXCL13 in MM-MΦ co-culture medium (Fig. 4E) and interfered with CXCL13 gene expression induction in MM cells co-incubated with macrophages (Fig. 4F). In addition, SB-431542 treatment partially abrogated the MM-promoted induction of CXCL13 gene expression in macrophages (Additional file 1: Fig. S3C). These results suggest that macrophage-provided signals that up-regulate CXCL13 expression in MM cells strongly rely on TGFβ.

### *CXCL13 regulates RANKL expression in BMSCs and MΦ and induces osteoclast (OC) formation *in vitro

**Fig. 5:**
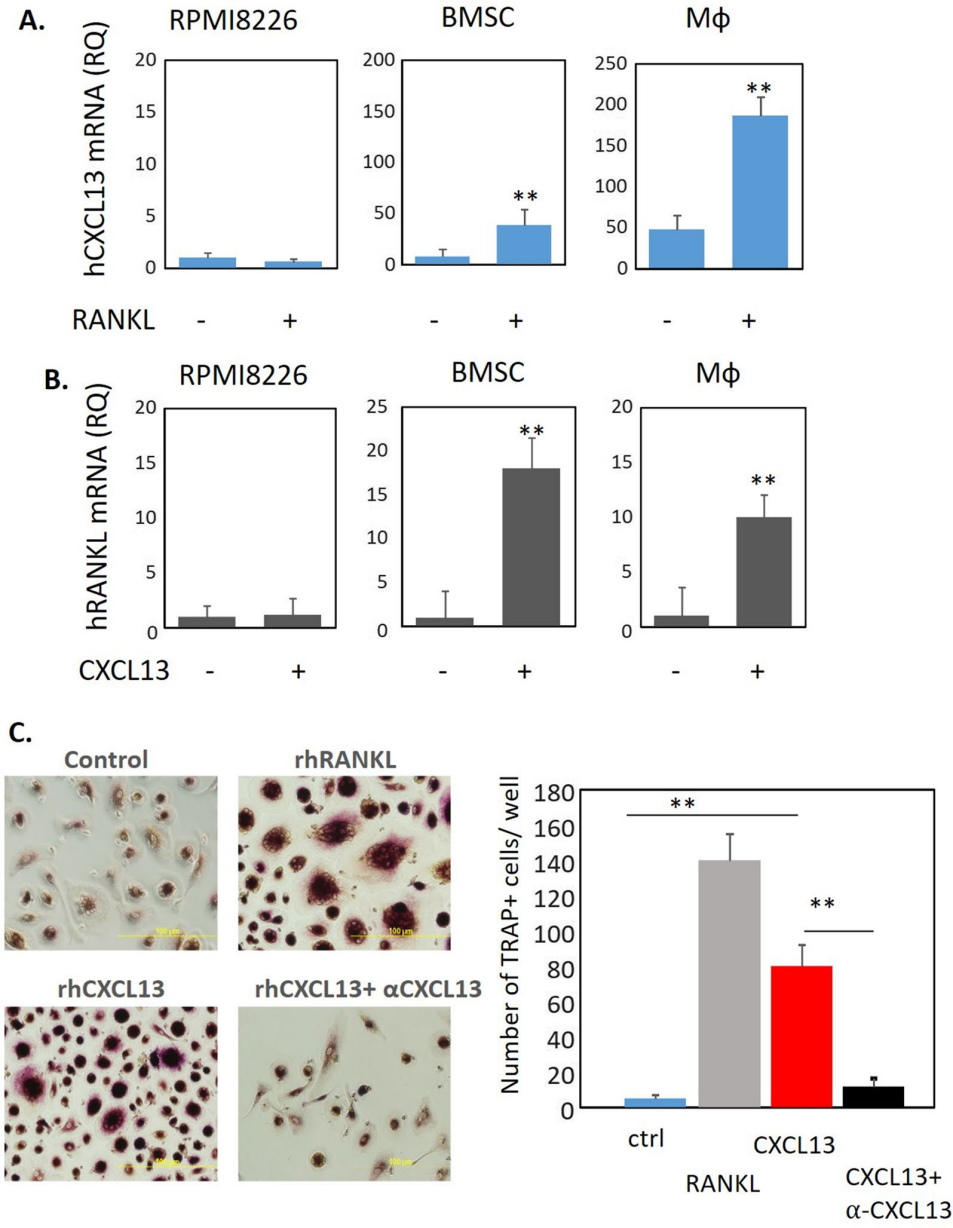
Reciprocal regulation of CXCL13 in RANKL in BMSC and Mф. Induction of osteoclast generation by CXCL13. RPMI8226 cells, primary BMSC or peripheral blood-generated macrophages were **A** treated with RANKL (100 ng/ml) for 48 h and mRNA level of CXCL13 was tested by qPCR, and **B** treated with CXCL13 (500 ng/ml) for 48 h and mRNA levels of RANKL was tested by qPCR. Data are presented as the mean of triplicates ± STDEV (**p < 0.01). **C** Human peripheral blood mononuclear cells from healthy donors were grown in the presence of recombinant human M-CSF (100 ng/ml) and RANKL (100 ng/ml) or CXCL13 (500 ng/ml) −/+ anti-CXCL13 (10 µg/ml) for 2 weeks. TRAP staining was performed and multinucleated TRAP+ cells were enumerated

Next, we evaluated the regulation and function of CXCL13. First, we demonstrated positive regulation feedback between CXCL13 and RANKL in BM stromal cells and macrophages, while such an effect could not be demonstrated in MM cells. Treatment with RANKL induced CXCL13 expression (Fig. 5A). Concomitantly, CXCL13 exposure induced RANKL mRNA levels in BMSC and MΦ (Fig. 5B). Furthermore, recombinant CXCL13 (100 ng/ml) induced in vitro formation of TRAP + OCs from PBMCs similar to the effect we observed with RANKL (50 ng/ml). Neutralizing antibodies against CXCL13 altered the ability of CXCL13 to promote OC formation (Fig. 5C). Of note, RANKL-induced CXCL13 up-regulation in MΦ was abrogated with ibrutinib treatment, suggesting the downstream involvement of BTK signaling pathway (Additional file 1: Fig. S4). Altogether, these results suggest the functional effects of elevated CXCL13 in the MM BM microenvironment and its role in osteoclast formation and activation.

### CXCL13 silencing in MM cells inhibits osteoclastogenesis and suppresses MM growth in vivo

**Fig. 6:**
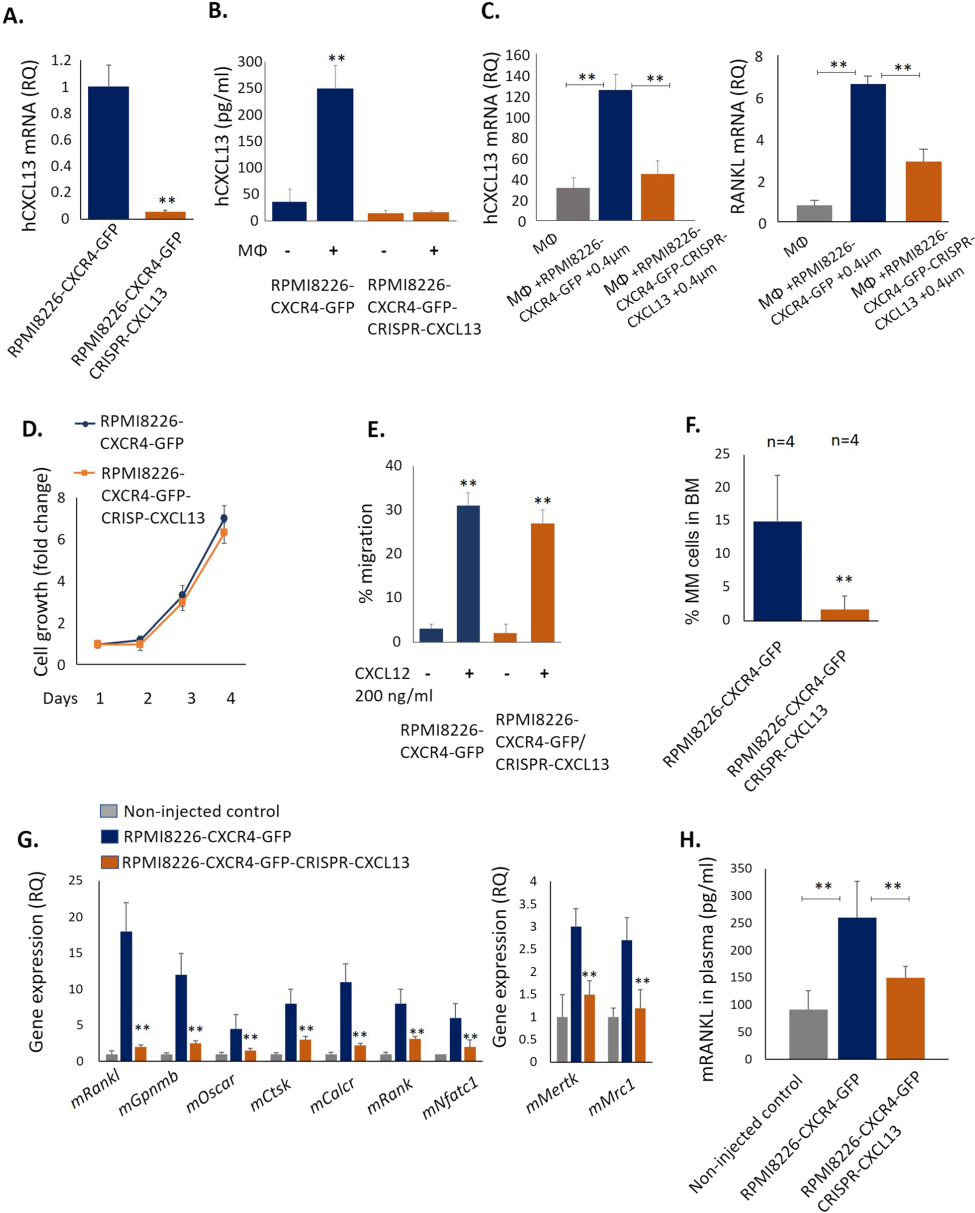
CXCL13 silencing in MM cells inhibits MM growth in vivo and suppresses MM-associated osteoclastogenesis in murine BM. **A** CXCL13 mRNA levels in RPMI8226-CXCR4-GFP and RPMI8226-CXCR4-GFP-CRISPR-CXCL13 cells evaluated by qRT-PCR. **B** CXCL13 secreted levels in conditioned medium of RPMI8226-CXCR4-GFP and RPMI8226-CXCR4-GFP-CRISPR-CXCL13 cells, cultured alone or co-cultured with peripheral-blood derived MΦ. **C** Peripheral-blood derived MΦ were cultured in the absence or presence of MM cell lines RPMI8226-CXCR4-GFP and RPMI8226-CXCR4-GFP-CRISPR-CXCL13, separated by 0.4 µm membrane for 48 h and subjected to subsequent analysis. Expression levels of CXCL13 and RANKL mRNA in MΦ cells evaluated by qRT-PCR. **D** In vitro cell growth kinetics of RPMI8226-CXCR4-GFP and RPMI8226-CXCR4-GFP-CRSIPR-CXCL13 cells, enumerated by FACS. **E** Transwell migration of RPMI8226-CXCR4-GFP and RPMI8226-CXCR4-GFP-CRSIPR-CXCL13 cells during 4 h in response to CXCL12 (200 ng/mL), evaluated by FACS. **F**-**H** NSG mice were i.v. inoculated with RPMI8226-CXCR4-GFP cells (*n* = 4) or RPMI8226-CXCR4-GFP-CRISPR-CXCL13 cells (*n* = 4). **F** MM tumor load was measured by enumeration of GFP+ human MM cells in the BM of inoculated animals. Data are presented as mean ± SE, **p < 0.01. **G** MNCs from the BM of control (*n* = 4) and MM-inoculated (*n* = 5) mice were purified and RNA was extracted. Gene expression of murine factors was evaluated by qRT-PCR. **H** Murine RANKL levels in plasma of non-inoculated (*n* = 3), RPMI8226-CXCR4-GFP (*n* = 4) and RPMI8226-CXCR4-GFP-CRISPR-CXCL13-inoculated (*n* = 4) animals at day 24 following cell injection. Data are presented as mean ± STDEV, **p < 0.01

To evaluate the role of CXCL13 in MM BM disease development, CXCL13 expression was silenced in CXCL13 in RPMI8226-CXCR4-GFP cells using CRISPR/Cas9 lentiviral transduction. Low CXCL13 mRNA expression levels were confirmed in RPMI8226-CXCR4-GFP CRISPR-CXCL13 cells (Fig. 6A). Furthermore, no further induction in CXCL13 secretion was observed upon co-incubation of CXCL13-silenced MM cells with macrophages (Fig. 6B). Notably, interaction with CXCL13-silenced MM cells induced significantly weaker induction of CXCL13 and RANKL expression in macrophages, in comparison with the original RPMI8226-CXCR4-GFP cells, suggesting the contribution of MM-secreted CXCL13 in CXCL13 and RANKL expression regulation in MΦ (Fig. 6C). Of note, CXCL13 silencing did not significantly affect in vitro growth of MM cells (Fig. 6D). Moreover, CXCL13 silencing did not interfere with CXCR4 activation in MM cells. RPMI8226-CXCR4-GFP-CRISPR-CXCL13 cells demonstrated effective trans-well migration in response to CXCL12 stimulation, comparable with the original RPMI8226-CXCR4-GFP cells (Fig. 6E). However, in vivo growth of CXCL13-silenced MM cells was significantly suppressed, reflected by decreased tumor burden in the BM of mice injected with RPMI8226-CXCR4-GFP CRISPR-CXCL13 in comparison with the parental RPMI8226-CXCR4-GFP cells (Fig. 6F). Further analysis of molecular markers associated with osteoclastogenesis and bone destruction revealed significant normalization and reduced expression levels of osteoclastogenic markers in the BM of mice inoculated with CXCL13-depleted MM cells. The presence of RPMI8226-CXCR4-GFP myeloma cells altered the BM milieu in favor of osteoclast genesis, inducing mRNA levels of murine osteoclast specific factors *Rankl*, *Gpnmb*, *Oscar*, *Ctsk*, *Calcr*, *Rank* and *Nfatc1*. In comparison, significantly reduced expression of osteoclast activation factors was observed in the BM of animals inoculated with CXCL13-silenced MM cells (Fig. 6G). Moreover, a decrease in the expression of markers associated with M2-type macrophage polarization (*Mrc1* and *Mertk*) was detected in the CXCL13-silenced group. Furthermore, plasma levels of circulating murine RANKL, well-known marker associated with osteoclast activation and bone destruction in MM [32], were significantly increased in tumor-bearing RPMI8226-CXCR4-GFP-inoculated animals compared to control non-injected and RPMI8226-CXCF4-GFP-CRISPR-CXCL13-inoculated mice (Fig. 6H). In accordance, RPMI8226-CXCR4-induced BM disease in mice promoted the loss of trabecular bone, while CXCL13 silencing in MM cells prevented it (Additional file 1: Fig. S6). These data suggest that high CXCL13 levels produced by MM cells affect the BM microenvironment, supporting MM proliferation, activating osteoclasts, and promoting M2 macrophage polarization.

### Elevated levels of CXCL13 in peripheral blood and bone marrow of MM patients associate with increased frequency of M2 macrophages in bone marrow of MM patients

**Fig. 7:**
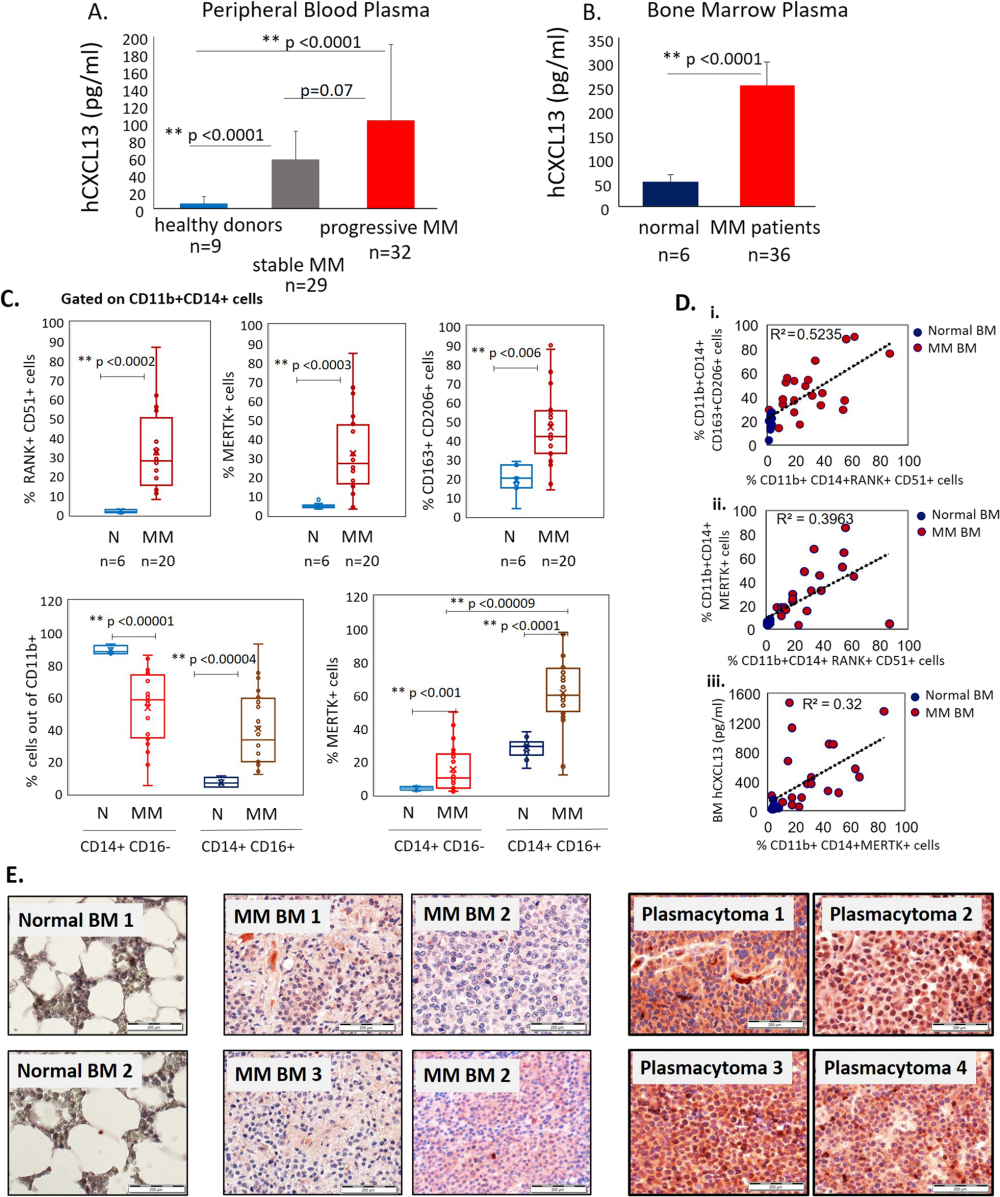
CXCL13 expression and flow cytometry analysis in primary MM samples. **A** CXCL13 levels in peripheral blood of MM patients with active disease (*n* = 32), stable disease (*n* = 29), or in healthy donors (*n* = 9), evaluated by ELISA. **B** CXCL13 levels in BM plasma samples of MM patients (*n* = 36) and healthy donors (*n* = 6), evaluated by ELISA. Data are presented as mean ± STDEV, **p < 0.01. **C** Flow cytometry analysis of cells in normal BM samples (*n* = 6) and MM BM samples (*n* = 20). The cells were pre-gated on CD11b+ CD14+ population. The frequency of RANK+ CD51+ cells, MERTK+ cells and CD163+ CD206+ was established (upper panel). The frequency of CD14+ CD16− and CD14+ CD16+ subpopulations, and percentage of MERTK+ cells in both subsets are presented (lower panel). **D** (i) Spearman’s rho correlation (two-tailed) between the frequency of CD163+ CD206+ and the frequency of RANK+ CD51+ subsets on CD11b+ CD14+ cells in BM samples (normal BM, *n* = 6; MM BM, *n* = 20). (ii) Spearman’s rho correlation (two-tailed) between the expression of MERTK and the co-expression of RANK and CD51 on CD11b+ CD14+ cells in BM samples (normal BM, *n* = 6; MM BM, *n* = 20). (iii) Spearman’s rho correlation (two-tailed) between CXCL13 protein levels in BM plasma and the frequency of MERTK expression on CD11b+ CD14+ cells in BM samples (normal BM, *n* = 6; MM BM, *n* = 20). **E** Expression of CXCL13 in normal BM samples, BM samples from patients with MM, and plasmacytoma samples, evaluated by immunohistochemical staining. Original magnification of × 400 is shown

Next, we evaluated the levels of CXCL13 chemokine in peripheral blood of MM patients (*n* = 61) and healthy donors (*n* = 9), using a commercially available ELISA kit. The patients’ clinical characteristics are summarized in Tables 1 and 2 (cohort 1). As demonstrated in Fig. 7A, plasma levels of CXCL13 were significantly higher in MM pts (148 pg/ml ± 136) in comparison with healthy individuals (19 pg/ml ± 7.6) (*p* < 0.001). Next, we evaluated the levels of CXCL13 in the BM plasma of MM patients (cohort 2). BM samples were collected from 36 newly diagnosed or relapsed MM patients. The patients’ clinical characteristics are summarized in Table 3. Additionally, BM plasma of healthy donors (*n* = 6) was collected as well. As depicted in Fig. 7B, significantly higher levels of CXCL13 (median 250 pg/ml, ± 55 pg/ml) were detected in MM patients compared to healthy controls (median 50 pg/ml, ± 22 pg/ml), *p* < 0.0001. Furthermore, CXCL13 BM levels were higher in comparison with peripheral blood levels in MM patients (cohort 1). These results suggest that elevated BM CXCL13 is associated with MM disease. Furthermore, the BM microenvironment represents a source of increased CXCL13 production in MM disease.

**Table 1:** Patient characteristics, cohort 1 (peripheral blood samples)

Characteristic	Stable MM disease (*n* = 29)	Progressive MM disease (*n* = 32)
Median age, years (range)	63.96 (39–84)	63.75 (32–82)
Gender		
Male	66% (19)	69% (22)
Female	34% (10)	31% (10)
Myeloma type		
IgG	52% (15)	50% (16)
IgA	17% (5)	19% (6)
Light chain only	24% (7)	15.5% (5)
Missing data	7% (2)	15.5% (5)
% MM cells in BM, range	40% (5–75%)	40% (10–75%)
Extramedullary disease	17% (5)	9.3% (3)
Bone lesions	48.3% (14)	59.3% (19)
Number of previous therapies (range)	2.5 (1–3)	3.5 (2–4)

MM, multiple myeloma; BM, bone marrow; IgG, immunoglobulin G; IgA, immunoglobulin A

**Table 2:** Laboratory and clinical parameters, cohort 1 (peripheral blood samples)

Characteristic	Stable MM disease (*n* = 29)	Progressive MM disease (*n* = 32)	*p* value
Hb	12.3	11.8	0.32
b2-mic	3.4	4.2	0.45
Albumin	4.13	3.88	0.21
LDH	163	180	0.7
IG	595	3782	0.005
Free light chain	72	186	0.29

Hb, hemoglobin; b2-mic, beta 2-microglobulin; IG, immunoglobulin; LDH, lactate dehydrogenase

**Table 3:** Patient characteristics, cohort 2 (bone marrow samples)

Characteristic	*n* = 36
Median age, years (range)	71 (52–92)
Gender	
Male	29% (11)
Female	69% (25)
Myeloma type	*n* = 33
IgG	51% (17)
IgA	3% (1)
Light chain only	
Kappa	36% (12)
Lambda	9% (3)
%MM cells in BM, range	40% (5–95%)
Bone lesions	22 (61%)
Number of previous therapies (range)	2.5 (1–3)

MM, multiple myeloma; BM, bone marrow; IgG, immunoglobulin G; IgA, immunoglobulin A

To investigate whether the elevated levels of CXCL13 observed in BM samples from MM patients could be associated with the increased numbers of M2 macrophages and osteoclast precursors, the immunophenotype of BM CD11b+ CD14+ cells was evaluated. The cumulative frequencies of CD51/RANK-double positive subset (defined as osteoclast precursors population), MERTK+ and CD163+ CD206+ M2 macrophage subsets were significantly increased in BM samples from MM patients (*n* = 20) in comparison with healthy donors (*n* = 6) (Fig. 7C, upper panel). Furthermore, significantly increased percentage of CD14+ CD16+ cells was found in MM BM samples compared to normal BM. Of note, MERTK expression predominantly characterized the CD14+ CD16+ fraction (Fig. 7C, lower panel). Notable, osteoclast precursor frequency (RANK + CD51+) correlated with CD163+ CD206+ (*R*^2^ = 0.5235) and MERTK+ (*R*^2^ = 0.3963) M2 macrophages subsets frequency (Fig. 7D(i, ii)) in BM samples. Moreover, the frequency of MERTK + M2 macrophages corresponded with CXCL13 BM levels (*R*^2^ = 0.32) (Fig. 7D(iii)).

Finally, immunohistochemical analysis of BM biopsies from MM pts (*n* = 10), plasmacytoma samples (*n* = 10) and normal BM samples (*n* = 2) demonstrated the expression of CXCL13 in malignant plasma cells. Notably, CXCL13 showed markedly increased expression within plasmacytoma tissues, suggesting that elevated CXCL13 levels may be associated with extramedullary disease (Fig. 7E).

## Discussion

Current evidence indicates that CXCL13 chemokine, originally discovered as a B-cell chemoattractant, is widely implicated in tumor development and progression [33]. CXCL13 was postulated to impact the proliferation, dissemination, and microenvironment interactions of several tumors, including solid cancers as well as hematological malignancies. Different cell types in the tumor milieu may overexpress CXCL13, including cancer cells, stromal and immune cells [34]. Dysregulated CXCL13 expression is seen in solid tumors including colorectal [35], breast [36], prostate [37], lung [38], and hepatocellular cancer [39] as well as lymphatic hematological malignancies like lymphoma [40] and CLL [20]. CXCL13 has been proposed as a biomarker and is included in the diagnostic criteria for angioimmunoblastic T-cell lymphoma (AITL), an aggressive nodal T cell lymphoma [41]. However, its role in MM remained largely unclear.

Our data highlight the role of CXCL13 in MM pathogenesis. Characterization of cross-talk mechanisms between malignant human myeloma cells and BM microenvironment in xenograft MM model identified an enrichment of CXCL13-expressing M2-macrophages in the BM infiltrated by MM. Furthermore, significantly elevated murine CXCL13 levels were detected in peripheral blood and BM of myeloma-inoculated animals and correlated with tumor burden. Further analysis revealed the upregulation of CXCL13 in the peripheral blood and BM of patients with MM, identifying both MM and stromal cells being sources of this aberrant CXCL13 overexpression. Moreover, CXCL13 expression was found to be strongly induced by the interactions between MM cell lines and microenvironment cells, including BM stromal cells and macrophages.

CXCL13 secretion by macrophages is known to be associated with M2 phenotype and tumor-promoting properties. For example, it was shown that CXCL13-secreting M2 macrophages in the tumor microenvironment promote the proliferation, invasion, and migration of renal cell carcinoma tumor cells [42]. Additionally, the relevance of CXCL13 expression in macrophages was implicated in lung cancer promoted by Benzo (a) pyrene (BaP), a carcinogen found in tobacco smoking. BaP-induced lung tumors in mice displayed increased CXCL13 levels, while CXCL13 or CXCR5 knockout significantly suppressed the development of Bap-induced lung cancer [43]. Importantly, CD68+ macrophages within the tumors were the origin of the observed increased CXCL13 levels [43]. Furthermore, a recent study identified CXCL13-secreting M2 macrophages being involved in premetastatic niche formation and promotion of colorectal liver metastasis [44]. In agreement, our results demonstrate increased frequency of MERTK-expressing M2 macrophages in BM of MM patients corresponding with elevated CXCL13 BM levels.

CXCL13 induction in MM BM milieu may further shape MM microenvironment composition in a paracrine way, recruiting various tumor-supporting immune cells. Accordingly, the role of CXCL13-CXCR5 cross talk in the recruitment and accumulation of myeloid-derived suppressor (MDSC) cells, inhibiting T cell expansion, and stimulating gastric cancer tumor growth was previously demonstrated [45]. Other studies revealed that B cells recruited by CXCL13 into prostate cancer tumors promote the progression of androgen-deprived prostate cancer leading to accelerated malignant progression and drug resistance [24, 46]. Increased CXCL13 within the tumor microenvironment may further facilitate immunosuppression due to increased recruitment of CXCR5-expressing regulatory T (Treg) cells [47].

In addition to immunosuppressive properties, CXCL13-expressing macrophages in the MM tumor microenvironment may be implicated in chemoresistance. The role of macrophages in the resistance and protection of MM cells from drug-induced apoptosis was previously demonstrated by others and us [30, 48]. Recent studies in MM cell lines recently show that BM mesenchymal stem cells from MM patients promote resistance to bortezomib in a CXCL13-dependent manner [49]. Therefore, our data provided a potential link between CXCL13 and tumor-supporting M2 macrophages in MM.

Mechanistically, we found that BTK signaling is involved in the macrophage’s CXCL13 up-regulation in response to MM-mediated stimulation, while BTK inhibition using ibrutinib effectively abrogated the increased CXCL13 production. As for the possible mechanism, BTK in macrophages can be activated through several pathways including chemokine signaling, Fc receptor signaling, and TLR signaling [50]. In accordance, our results identify TLR4 signaling upstream to BTK being involved in MM-mediated induction of CXCL13 in macrophages, since MYD88 inhibition effectively abrogated the increase in CXCL13 expression. BTK signaling is known to be tightly interconnected to CXCL13 activity. Early studies have shown that BTK inhibition suppressed CXCL13-induced signaling, adhesion, and migration of primary CLL cells [51, 52]. Furthermore, treatment with ibrutinib has been shown to reduce CXCL13 levels in patients with CLL and Waldenstrom macroglobulinemia (WM) [53, 54]. Finally, CXCL13 was found to be a robust predictive marker of major response to ibrutinib in WM patients [54]. Our data suggest that BTK activation may regulate CXCL13 expression in macrophages in the MM microenvironment, identifying BTK as a potential therapeutic target in the MM milieu. Although the therapeutic effect of ibrutinib in MM is clinically less satisfying than in other lymphatic hematological malignancies still it may have a role in a selected group of MM patients as was demonstrated in a clinical trial by Richardson P. and colleagues that were able to show that in heavily treated population with relapsed/refractory MM patients having received a median of 4 prior treatments, ibrutinib in combination with dexamethasone still produced encouraging responses [55]. Dexamethasone is known to induce M2c polarization in macrophages [56]. Therefore, the beneficial effect of ibrutinib in combination with dexamethasone may partially be attributed to the ability of the BTK inhibitor to suppress dexamethasone-induced M2c polarization and an associated increase in CXCL13 in the MM niche.

A distinct molecular mechanism is implicated in macrophage-mediated up-regulation of CXCL13 in MM cells. CXCL13 induction in MM cells upon the co-culture with macrophages is driven by TGFβ signaling. Our results demonstrate that the TGFβ production by macrophages promotes CXCL13 expression in MM cells, while inhibition of TGFβ receptors interfered with the CXCL13 up-regulation. These results aligned with data from previous studies showing the interconnection between TGFβ signaling and CXCL13 regulation and expression. TGFβ was shown to induce CXCL13 expression in PD-1^high^CD4^+^ cells under inflammatory conditions and in cancer [57, 58]. Moreover, stimulation with TGFβ induced CXCL13 secretion in CD8^+^ cells and increased CD103+ CD8+ tumor-infiltrating T cell subpopulation produced CXCL13 in ovarian and uterine cancers [59]. Conversely, inhibition of TGFβ receptors abrogated CXCL13 signaling in the tumor-associated T cells [59]. TGFβ is a vital factor in the pathogenesis of MM. TGFβ is elaborated by myeloma cells, immune cells, and BM stromal cells, stimulating IL-6 and VEGF secretion, promoting angiogenesis, and supporting myeloma progression [60, 61]. Furthermore, TGFβ activity is associated with lytic bone disease in MM [60–62]. Here, our recent data determine the role of stroma-produced TGFβ in CXCL13 up-regulation in the MM milieu and suggest that TGFβ blockade using novel inhibitors may result in CXCL13 suppression and BM microenvironment normalization.

Current results may also indicate that increased CXCL13 in the MM BM milieu can be involved in bone invasion and extramedullary disease. Increased CXCL13 production and release from MM and macrophages may contribute to augmented RANKL expression in the stromal compartment, therefore ultimately resulting in osteoclast generation. In support, we detected reciprocal regulation between RANKL and CXCL13 in stromal cells. Notably, RANKL-induced CXCL13 up-regulation in macrophages was BTK dependent. To notice, we observed that CXCL13 inhibition by neutralizing antibodies effectively decreased osteoclast formation in vitro. Furthermore, CXCL13 silencing using CRISPR editing in MM cells suppressed MM growth in the BM niche in vivo and significantly reduced the expression of osteoclastogenic markers and trabecular bone destruction in the BM of mice inoculated with CXCL13-depleted MM cells. Accordingly, conditioned medium produced by CXCL13-depleted MM cells did not increase CXCL13 and RANKL expression in the macrophages, supporting the regulatory role of CXCL13 produced by MM cells in osteoclastogenic environment generation. These findings are in agreement with previous studies identifying the role of CXCL13 in osteoclastogenesis and bone resorption. CXCL13 was shown to significantly enhance the RANKL signaling pathway and osteolysis in oral squamous cell carcinoma (OSCC) [63, 64]. A subsequent study identified c-Myc activation to be involved in CXCL13-mediated upregulation of RANKL in tumor bone marrow environment in the OSCC [65]. Our results reveal the possible role of elevated CXCL13 in MM-associated osteoclastogenesis, suggesting that CXCL13 targeting may prevent osteoclast growth and bone destruction in MM patients.

Importantly, therapeutic modalities targeting CXCL13-CXCR5 signaling in the tumor microenvironment are currently being explored, although no small molecule pharmacological inhibitors directly targeting CXCL13 or CXCR5 have been yet developed. However, gene knockdown evidence including the neutralization of overexpressed CXCL13 or CXCR5 activities has displayed the therapeutic potential of this important chemokine axis in numerous cancers [25, 43, 66]. At the moment, various novel approaches are being employed to inhibit CXCL13/CXCR5 signaling in the tumor microenvironment and our study is in line and further support this effort by providing additional valuable information.

## Conclusions

In sum, our study discloses the importance of CXCL13 in MM, indicating a complex network of cellular interactions involving elaborated CXCL13 in MM progression. These findings contribute to our global understanding of the microenvironment’s role in MM and identify CXCL13 as a possible novel therapeutic target for MM. CXCL13 blockade might be a promising strategy to suppress osteoclast activation and MM progression.

## Supplementary Information

**Additional file 1.** Supplementary methods and results.

## Data Availability

Not applicable.

## Abbreviations

AEC: 3-Amino-9-ethyl carbazole
ATCC: American type culture collection
BTK: Bruton’s tyrosine kinase
BM: Bone marrow
BMSC: Bone marrow stromal cells
CFSE: Carboxyfluorescein succinimidyl ester
CLL: Chronic lymphocytic leukemia
CXCL13: C-X-C motif chemokine ligand 13
CXCR4: C-X-C motif chemokine receptor 4
CXCR5: C-X-C motif chemokine receptor 5
DNA: Deoxyribonucleic acid
EDTA: Ethylenediaminetetraacetic acid
ECL: Enhanced luminol-based chemiluminescent
ELISA: Enzyme-bound immunosorbent analysis
Erk 1/2: Extracellular signal-regulated protein kinase 1/2
FACS: Fluorescence-activated cell sorting
FCS: Fetal calf serum
FITC: Fluorescein isothiocyanate
GFP: Green fluorescent protein
GPNMB: Glycoprotein nonmetastatic melanoma protein B
IMiDs: Immunomodulatory drugs
JNK: C-Jun N-terminal kinase
qRT-PCR: Quantitative reverse transcription polymerase chain reaction
LPS: Lipopolysaccharide
M-CSF: Macrophage colony-stimulating factor
MM: Multiple myeloma
MERTK: MER proto-oncogene, tyrosine kinase
MYD88: Myeloid differentiation primary response 88
MΦ: Macrophages
OC: Osteoclast
OSCAR: Osteoclast associated Ig-like receptor
PI: Propidium iodide
PVDF: Polyvinylidene fluoride
SDS-PAGE: Sodium dodecyl sulfate–polyacrylamide gel electrophoresis
STAT3: Signal transducer and activator of transcription 3
STDEV: Standard deviation
STR: Short tandem repeat
TGFβ: Transforming growth factor *β*
TLR: Toll-like receptor
Treg: Regulatory T
TRAP: Triiodothyronine receptor auxiliary protein
RANKL: Receptor activator of nuclear factor kappa-B ligand
RNA: Ribonucleic acid
