# Supplementary material for: CXCL13 chemokine is a novel player in multiple myeloma osteolytic microenvironment, M2 macrophage polarization, and tumor progression
Source: J Hematol Oncol. 2022 Oct 10;15:144. doi: 10.1186/s13045-022-01366-5 (PMC9549634; doi:10.1186/s13045-022-01366-5)
Supplement: Supplementary file 1 — Additional file 1. Supplementary methods and results. [file 13045_2022_1366_MOESM1_ESM.docx]

**Additional file 1. Supplementary Methods and Results.**

**Additional Methods**

**Additional Table 1. Primers sequences.**

| Gene | Forward | Reverse |
| --- | --- | --- |
| Human beta2-microglobulin | AGGCTATCCAGCGTACTCCA | TCAATGTCGGATGGATGAAA |
| Human CXCL13 | CTCTGCTTCTCATGCTGCTG | TCGATCAATGAAGCGTCTAGG |
| Human RANKL | TCGTTGGATCACAGCACATCA | TATGGGAACCAGATGGGATGTC |
| Human TGFβ | GTACCTGAACCCGTGTTGCT | GAACCCGTTGATGTCCACTT |
| Mouse HPRT | GGACCTCTCGAAGTGTTGGATAC | GCTCATCTTAGGCTTTGTATTTGGCT |
| Mouse Cxcl13 | AACTCCACCTCCAGGCAGAATG | TGTGTAATGGGCTTCCAGAATACC |
| Mouse Rankl | TGGAAGGCTCATGGTTGGAT | CATTGATGGTGAGGTGTGCA |
| Mouse Gpnmb | TCTGAACCGAGCCCTGACATC | AGCAGTAGCGGCCATGTGAAG |
| Mouse Oscar | TGGCGGTTTGCACTCTTCA | GATCCGTTACCAGCAGTTCCAGA |
| Mouse Ctsk | GGGCCAGGATGAAAGTTGTA | CACTGCTCTCTTCAGGGCTT |
| Mouse Calcr | CTTCCATGCTGATCTTCTGG | CAGATCTCCATTGGGCACAA |
| Mouse Rank | GCATCCCTTGCAGCTCAACA | ATGGAAGAGCTGCAGACCAC |
| Mouse Nfatc1 | TGAGGCTGGTCTTCCGAGTT | CGCTGGGAACACTCGATAGG |
| Mouse Mertk | GCAGGGACTTACAAAGAGCTTTC | AGCCGAGGATGATGAACATAGAG |
| Mouse CD206 (Mrc1) | ATGGATTGCCCTGAACAGCA | TGTACCGCACCCTCCATCTA |

**Additional Table 2. Flow cytometry antibodies.**

| Antibody | Fluorochrome | Clone | Provider | Dilution |
| --- | --- | --- | --- | --- |
| Mouse CD11b | PE | M1/70 | Biolegend | 1:100 |
| Mouse F4/80 | PE-Cy7 | BM8 | Biolegend | 1:100 |
| Mouse CD206 | APC | C068C2 | Biolegend | 1:100 |
| Mouse MERTK | APC | 2B10C42 | Biolegend | 1:100 |
| Human CD11b | PE | LM2 | Biolegend | 1:100 |
| Human CD163 | PerCP-Cy5.5 | GHI/61 | Biolegend | 1:100 |
| Human CD206 | APC | 15-2 | Biolegend | 1:100 |
| Human MERTK | PE-Cy7 | 590H11G1E3 | Biolegend | 1:100 |
| Human MERTK  Human CD14  Human CD16  Human RANK  Human CD51 | AlexaFluor 647  PE-Cy7  FITC  AlexaFluor 488  APC | 590H11G1E3  M5E2  3G8  NKI-M9 | Biolegend  Biolegend  Biolegend  R&D Systems  Biolegend | 1:100  1:100  1:100  1:100  1:100 |

**Additional Figure 1. CXCL13 up-regulation in MM cells upon interaction with macrophages is not affected by BTK inhibition.** MM cells RPMI8226 and CAG were cultured in the absence or presence of peripheral-blood-derived MΦ, with or without ibrutinib (20 µM) for 48 hours. CXCL13 mRNA levels in MM cells were evaluated by qRT-PCR. Data are presented as mean of triplicates ±STDEV (**p<0.01).

**Additional Figure 2.** Peripheral-blood derived MΦ were cultured in the absence or presence of MM cells RPMI8226, separated by 0.4 µm membrane with or without LPS (500 ng/mL), ST2825 (10 µM) and ibrutinib (20 µM) for 48 hours and subjected to subsequent analysis. (A) Expression levels of CXCL13 mRNA in MΦ cells was evaluated by qRT-PCR. (B) Levels of secreted CXCL13 in the culture medium were evaluated by ELISA. Data are presented as the mean of triplicates ±STDEV (**p<0.01).

**Additional Figure 3.** **TGFβ is expressed in both MM and macrophages and is up-regulated upon their interaction.** (A) Peripheral-blood-derived MΦ were cultured in the absence or presence of MM cell lines RPMI8226 or CAG (direct co-culture) for 48 hours. Levels of secreted TGFβ in the culture medium were evaluated by ELISA. Data are presented as the mean of triplicates ±STDEV (**p<0.01). (B) TGFβ mRNA expression in peripheral-blood generated MΦ, cultured in the absence or presence of RPMI8226 and CAG cells separated by 0.4 µm membrane, evaluated by qRT-PCR. Data are presented as the mean of triplicates ±STDEV (**p<0.01). (C)

**Additional figure 4. RANKL-induced up-regulation of CXCL13 in MΦ involves BTK signaling.** Peripheral-blood-derived MΦ were treated with RANKL (100 ng/ml), ibrutinib (20 µM) or their combination for 48h and mRNA level of CXCL13 was tested by qPCR. Data are presented as the mean of triplicates ±STDEV (**p<0.01).

**Additional Figure 5. BM samples characterization.** (A) Frequency of RANK^+^ CD51^+^ osteoclast precursors in normal BM samples (n=6) and MM BM samples (n-20) evaluated by flow cytometry. Representative flow cytometry dot plots examining the proportion of RANK^+^ CD51+ subset in CD11b^+^ CD14^+^ population. The cells were pre-gated on CD11b^+^ CD14^+^ population. (B) Frequency of MERTK^+^ M2 MΦ precursors in normal BM samples (n=6) and MM BM samples (n-20). Representative flow cytometry dot plots for gating strategy evaluating the expression of murine CD11b, CD14, CD16, and MERTK markers. The cells were pre-gated on CD11b^+^ CD14^+^ population. MERTK expression in CD14^+^CD16^-^ and CD14^+^CD16^+^ populations was evaluated.

**Additional Figure 6. RPMI8226-CXCR4-induced disease in mice promotes the loss of trabecular bone. CXCL13 silencing in MM cells prevents the development of osteolysis in mice.** Histochemical analysis of the femurs in representative animals. Low-magnification (x4) images of the trabecular bone (blue arrows) in femurs from the non-injected, RPMI8226-CXCR4-injected and RPMI8226-CXCR4-CRISPR-CXCL13-injected mice are presented.
